# Occurrence of passion fruit woodiness disease in the coastal lowlands of Kenya and screening of passion fruit genotypes for resistance to passion fruit woodiness disease

**DOI:** 10.1186/s12870-023-04546-8

**Published:** 2023-11-06

**Authors:** Lydia K. Asande, Omwoyo Ombori, Richard O. Oduor, Shem B. Nchore, Evans N. Nyaboga

**Affiliations:** 1 Department of Plant Science, Kenyatta University, P.O. Box 43844 – 00100, Nairobi, Kenya; 2 Department of Biochemistry, University of Nairobi, P.O. Box 30197 – 00100, Nairobi, Kenya; 3 Department of Biochemistry and Biotechnology, Kenyatta University, P.O. Box 43844 – 00100, Nairobi, Kenya

**Keywords:** Passiflora, Passion fruit woodiness disease, Viruses, Disease resistance

## Abstract

**Background:**

Passion fruit (*Passiflora edulis* [Sims]) is an important economic fruit crop in Kenya, grown for domestic, regional and international markets. However, passion fruit production is constrained by both biotic and abiotic stresses. Passion fruit woodiness disease (PWD) complex is the most injurious viral disease responsible for yield losses of up to 100%. In East Africa*,* it is caused by potyviruses. The most effective way to manage PWD is by using resistant cultivars. The objectives of this study were to determine the occurrence of passion fruit woodiness disease in selected counties at the Coastal lowlands of Kenya and screen farmer preferred passion fruit genotypes for resistance to PWD.

**Results:**

In the present study, it was established that all surveyed farms in Kwale and Kilifi counties displayed passion fruit woodiness virus disease symptoms. The highest disease incidence of 59.16% and 51.43% was observed at Kilifi and Kwale counties, respectively. A significant difference (*p* < 0.05) in symptom severity was observed within the tested genotypes with purple and banana passion fruits having the highest and lowest AUDPC values, respectively, both under greenhouse and field conditions. ACP ELISA assays using universal potyvirus antiserum (Agdia Inc., Elkhat, IN) confirmed that the observed characteristic symptoms of woodiness disease were as a result of potyvirus infection.

**Conclusions:**

The findings herein indicate that PWD is widespread in both Kilifi and Kwale counties with low to moderate disease incidence and severity. The observed prevalence, incidence and severity levels of PWD in Kwale and Kilifi counties could be aggravated by poor management practices such as non-sterilization of pruning tools, intercropping with target crops and crop rotation with the same target crops. Response of passion fruit genotypes to woodiness viruses was genotype dependent. There is need to sensitize farmers on the cause and spread of PWD and management strategies in order to increase production and enhance the quality of fruits.

**Supplementary Information:**

The online version contains supplementary material available at 10.1186/s12870-023-04546-8.

## Background

Passion fruit (*Passiflora edulis* Sims) is a commercially important tropical fruit crop of the family *Passifloraceae,* mainly cultivated in the tropical and subtropical countries, for its nutritional, medicinal, ornamental and cosmetic value [1]. The vigorous vine is native to tropical America and extensively cultivated in Brazil [2]. In Kenya, passion fruit flourishes in a vast array of altitudes of up to 2,000 m a. s. l. [3]. Two different types of *Passiflora edulis* Sims are predominantly cultivated in Kenya: the purple type which thrives at higher altitudes (18—25°C) and the yellow type, which flourishes in the tropical lowlands (25- 30°C).

The world’s total production of passion fruit is about 1.5 million tonnes. Brazil is the world’s largest producer generating about 90% of the total production. In Kenya, passion fruit is a major constituent of the horticulture industry for local and export markets that provides an invaluable source of income to several households [4]. The fruit is ranked fourth amongst fruit exports in Kenya [5]. Furthermore, it has a huge commercial potential due to the increasing demand for fresh fruit, processed juice and the booming export markets [6]. In 2020, passion fruit generated USD 14.5 million from 41,879 MT produced on an area of 3,322 Ha [7]. However, in spite of the great potential of passion fruit, production has dwindled to a mean yield of 12 ton ha^−1^ against a prospective yield of 24 ton ha^−1^ [4, 7] which can largely be attributed to pests, diseases and scarcity of disease-free planting materials [6, 7]. Additionally, these restraints have reduced the lifetime of passion fruit in Kenya from 7 years [8] to a mean of 1 to 2 years [4].

Passion fruit woodiness disease is one of the major biotic stresses constraining production. This disease is largely spread by aphids across areas of production and it affects genotypes predominantly grown in Kenya [9], including yellow and purple genotypes [10]. It can be detrimental causing massive losses of up to 100% [11]. The method of propagation which involves usage of seeds and grafting causes accumulation especially of passion fruit woodiness virus complex occasioning massive fruit losses. The control and management of PWD is ordinarily problematic and often not possible, largely due to the non-persistent transmission of viruses by numerous aphid vectors. Furthermore, *Cowpea Aphid Borne Mosaic Virus* (CABMV) and *Passion Fruit woodiness virus* (PWV) have numerous wild hosts serving as reservoirs for the viruses [12]. In Kenya, different isolates of CABMV have been reported as the primary causal agents of woodiness disease [13, 14]. However, the isolates have minimal variability [13]. Therefore, it is imperative to control passion fruit woodiness disease to scale up production or at least sustain reasonable yields and safeguard susceptible varieties from total loss. Varieties resistant to passion fruit woodiness disease can be effective for control of the viruses.

The success of disease management strategies is dependent on sufficient information on the prevalence, incidence, severity and awareness amongst farmers in affected regions. Although passion fruit woodiness disease is a major challenge in passion fruit growing locations in Western and Eastern Kenya [15], no survey has been reported in the coastal lowlands of Kenya to establish disease occurrence, distribution and severity. Moreover, there have been lamentations from farmers reporting incidences of pests and diseases that devastate passion fruit orchards [15]. The present survey was vital in the advancement of proper and sustainable disease management measures in the coastal lowlands of Kenya.

The objectives of this study were to determine the prevalence, incidence and severity of passion fruit woodiness disease in the coastal lowlands of Kenya and determine resistance of selected passion fruit genotypes to woodiness disease under greenhouse and field conditions.

Findings on the occurrence of passion fruit woodiness disease in the coastal lowlands of Kenya would impact positively in the mitigation measures to improve productivity of passion fruits. Screening for resistance to woodiness disease is correspondingly indispensable in the identification of promising genotypes for integration into future breeding program to mitigate the damage caused by this disease.

## Results

### Prevalence, incidence and severity of passion fruit woodiness disease in Kilifi and Kwale counties

All surveyed farms in Kwale county displayed virus disease symptoms (disease prevalence of 100%). Virus disease incidence ranged from 32.50% to 51.43%, with an overall mean of 43.27%. The highest disease incidence was recorded in Mivumoni (51.43%) and the lowest in Mangawani (32.50%). Mean disease severity ranged from 2.40 to 2.77 across the six locations, with an overall mean of 2.66. The highest PWD severity (2.77) was scored in farmers’ fields in Lukore location while the lowest was in Shimba hills (2.40) (Table 1). The mean disease severity differed significantly (*P* ≤ 0.05) between orchards surveyed in Shimba hills, and those in Mivumoni and Lukore.

**Table 1:** Prevalence, incidence and severity of passion fruit woodiness disease (PWD) in Kwale County, Kenya

Location	Sample size	Prevalence of PWD (%)	PWD incidence (%)^c^	Severity of PWD^c^ (Mean ± S.E)
Lukore	7	100	47.14 ± 4.48^ab^	2.77 ± 0.07^a^
Mwaluvanga	5	100	35.00 ± 3.53^b^	2.59 ± 0.09^ab^
Mivumoni	7	100	51.43 ± 4.46^a^	2.75 ± 0.07^a^
Shimba hills	5	100	47.00 ± 6.63^ab^	2.40 ± 0.08^b^
Mangawani	8	100	32.50 ± 2.83^b^	2.64 ± 0.08^ab^
Manyatta	8	100	48.12 ± 3.65^ab^	2.65 ± 0.07^ab^
Mean total		100	43.27	2.66

^c^Values denote mean ± standard error of 20 replicates per orchard. Means having identical letters within a column were not significantly different according to Tukey’s HSD test at 5% level

All surveyed farms in Kilifi County were infected with PWD with 100% disease prevalence (Table 2). Virus disease incidence ranged from 33.75% to 59.16% (Table 2) with overall mean of 46.00% across the 8 locations. Disease severity ranged from 2.65 to 3.18. The highest disease incidence (59.16%) and disease severity (3.18%) was recorded in Ganda, while the least disease incidence was recorded in Mida (Table 2). The disease severity was significantly higher (*P* ≤ 0.05) in the orchards surveyed in Ganda compared with those from Mbaraka Chembe. However, there was no significant difference established between the other orchards. Ganda had both KPF 4 and yellow variety of passion fruits varieties. Kilifi county displayed a higher mean disease incidence (46.00) and severity (2.96) compared to Kwale county (43.27 and 2.66, respectively).

**Table 2:** Prevalence, incidence and severity of passion fruit woodiness disease (PWD) in Kilifi County, Kenya

Location	Sample size	Prevalence of PWD (%)	PWD Incidence (%)^c^	Severity of PWD^c^ (Mean ± S.E)
Mbaraka Chembe	4	100	42.50 ± 3.22^ab^	2.65 ± 0.11^b^
Mkenge	8	100	44.37 ± 2.39^ab^	2.97 ± 0.07^ab^
Ganda	6	100	59.16 ± 8.28^a^	3.18 ± 0.08^a^
Kijiwetanga	7	100	45.71 ± 2.77^ab^	2.91 ± 0.08^ab^
Mida	4	100	33.75 ± 3.15^b^	2.73 ± 0.14^ab^
Dabaso	3	100	48.57 ± 8.82^ab^	2.89 ± 0.12^ab^
Goshi	3	100	36.66 ± 4.41^ab^	2.82 ± 0.14^ab^
Mtwapa	5	100	50.00 ± 5.70^ab^	3.08 ± 0.09^ab^
Mean total		100	46.00	2.96

^c^Values denote mean ± standard error of 20 replicates per orchard. Means having identical letters within a column were not significantly different according to Tukey’s HSD test at 5% level

### Disease symptoms observed on plants and fruits in the field

**Fig. 1:**
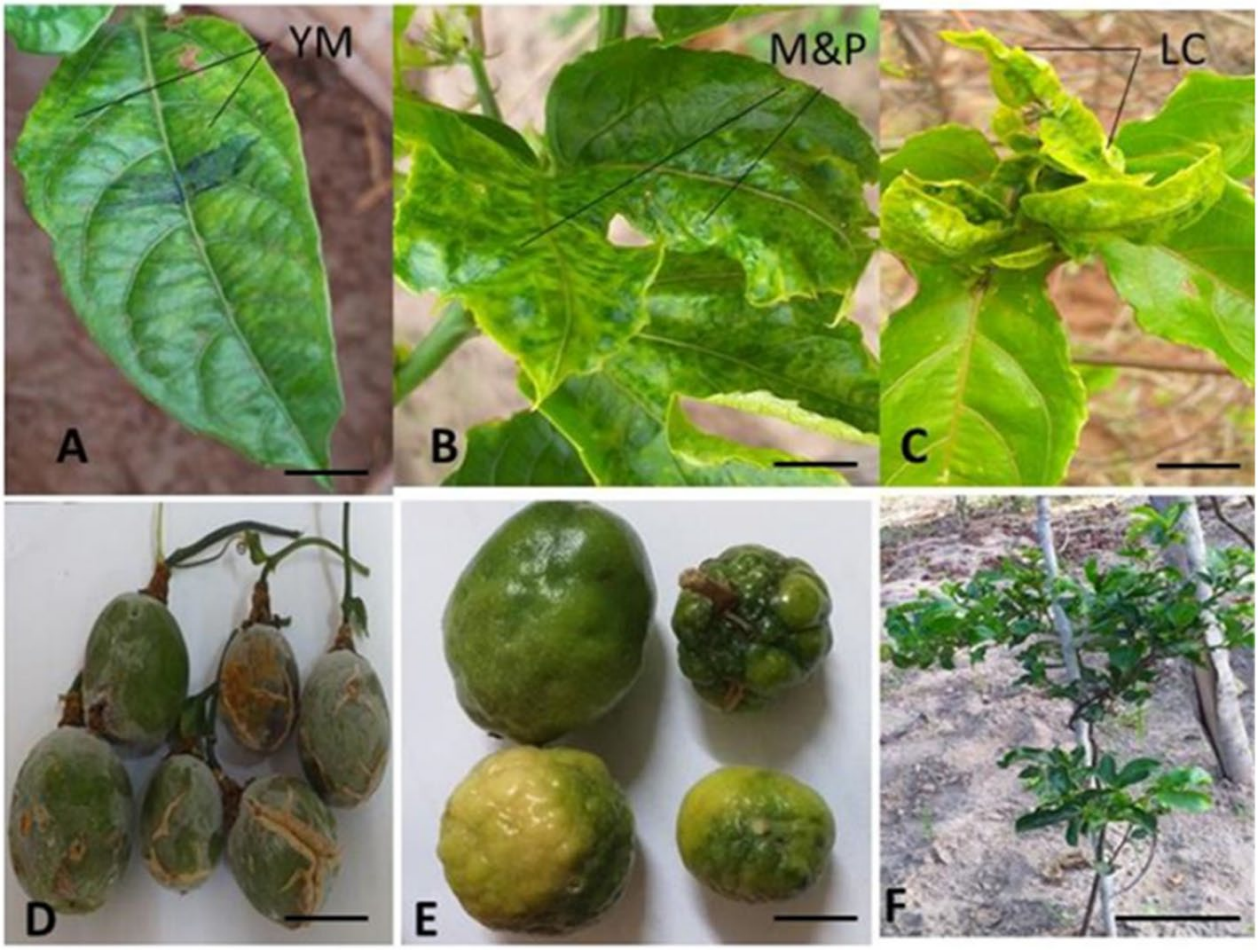
Symptoms of passion fruit woodiness disease observed in the field. **A**, Yellow leaf mosaic (YM) (Bar = 2 cm); **B**, Leaf mottling with puckering (M&P) (Bar = 2 cm), **C**, Leaf curl (LC) (Bar = 2 cm); **D**, Purple passion misshapen fruits with a corky rind (Bar = 2 cm); **E**, Yellow passion misshapen fruits with a corky rind (Bar = 2 cm), **F**, Three year old stunted plant (Bar = 10 cm)

Virus disease symptoms were recorded in all the surveyed farms in both Kwale and Kilifi counties. The plants exhibited characteristic symptoms of woodiness disease such as yellow leaf mosaic, leaf mottling, leaf puckering, distortion coupled with reduction in size and leaf curl (Fig. 1A, B and C). The fruits were deformed, with unusually corky rinds and reduced in size (Fig. 1D and E). In severe cases, affected plants displayed stunted growth (Fig. 1F).

### Passion fruit woodiness disease management practices

A total of 45% and 55% farmers in Kwale and Kilifi counties respectively were able to identify woodiness disease symptoms. However, there was no statistical association between management practices and the different locations within Kwale and Kilifi counties (Table 3).

**Table 3:** Test of association between woodiness disease management practices and locations in Kwale and Kilifi counties

Kwale County		Kilifi County	
Location	Pearson Chi-square	Location	Pearson Chi-square
Lukore	χ^2^ = 2, *P* = 0.849	Mbaraka Chembe	χ^2^ = 3.778, *P* = 0.707
Mwaluvanga	χ^2^ = 1, *P* = 0.963	Mkenge	χ^2^ = 4, *P* = 0.677
Mivumoni	χ^2^ = 8.5, *P* = 0.131	Ganda	χ^2^ = 3.852, *P* = 0.697
Shimba hills	χ^2^ = 2.556, *P* = 0.635	Kijiwetanga	χ^2^ = 6.966, *P* = 0.324
Mangawani	χ^2^ = 5.194, *P* = 0.393	Mida	χ^2^ = 1.263, *P* = 0.974
Manyatta	χ^2^ = 9.077, *P* = 0.106	Dabaso	χ^2^ = 2.267, *P* = 0.894
		Goshi	χ^2^ = 2.73, *P* = 0.965
		Mtwapa	χ^2^ = 6, *P* = 0.423

**Fig. 2:**
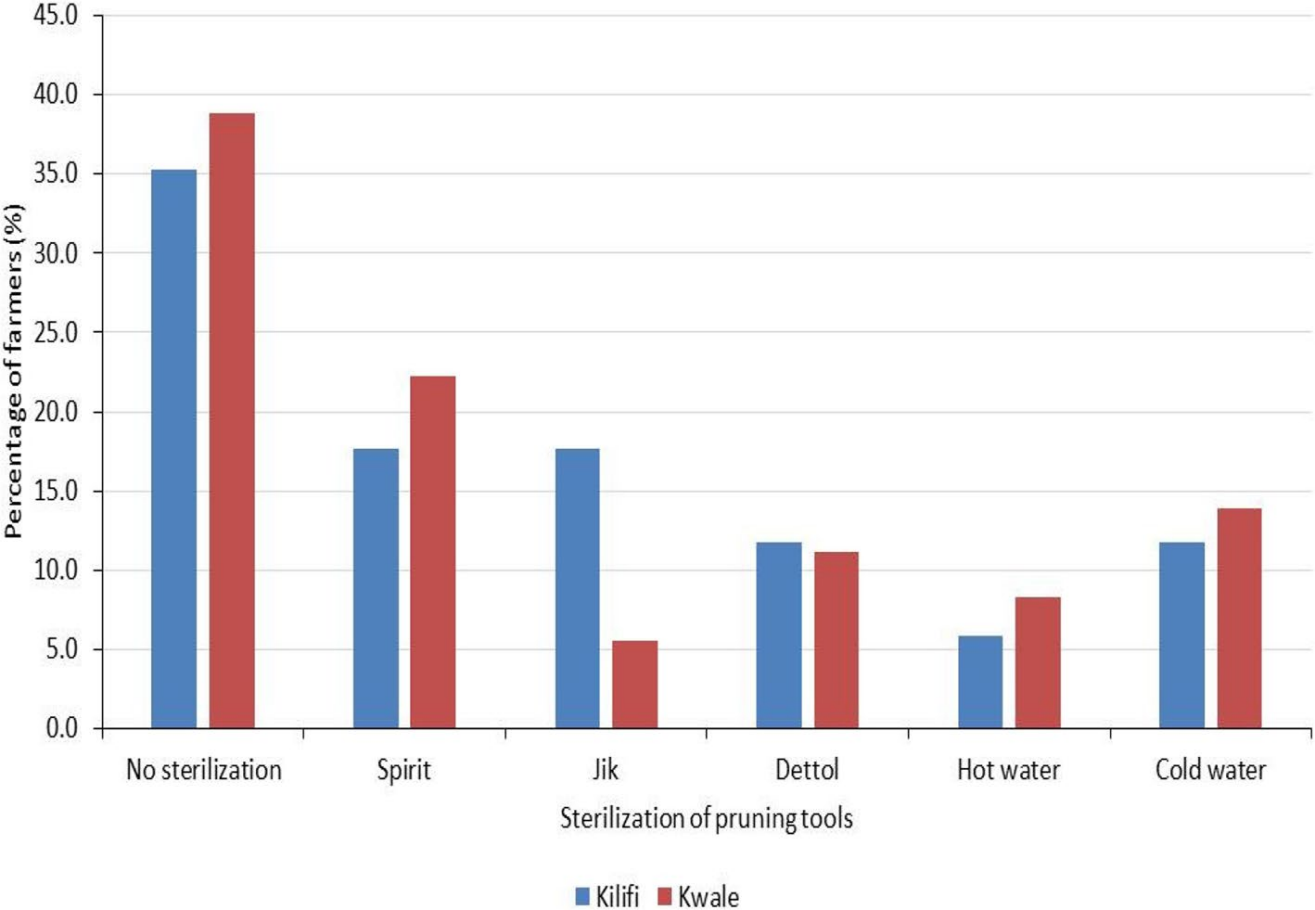
Percentage of farmers using different methods of sterilization of pruning tools in Kwale and Kilifi counties

The highest percentage of farmers in Kilifi and Kwale counties (35.3% and 38.9% respectively) did not sterilize their pruning tools (Fig. 2). However, majority of the farmers in Kilifi county sterilized their pruning tools using methylated spirit and jik, while in Kwale county, a higher proportion of farmers sterilized their pruning tools using methylated spirit. The method of sterilization did not differ significantly (χ^2^ = 4.181, *P* > 0.05) in the two counties (Fig. 2).

**Fig. 3:**
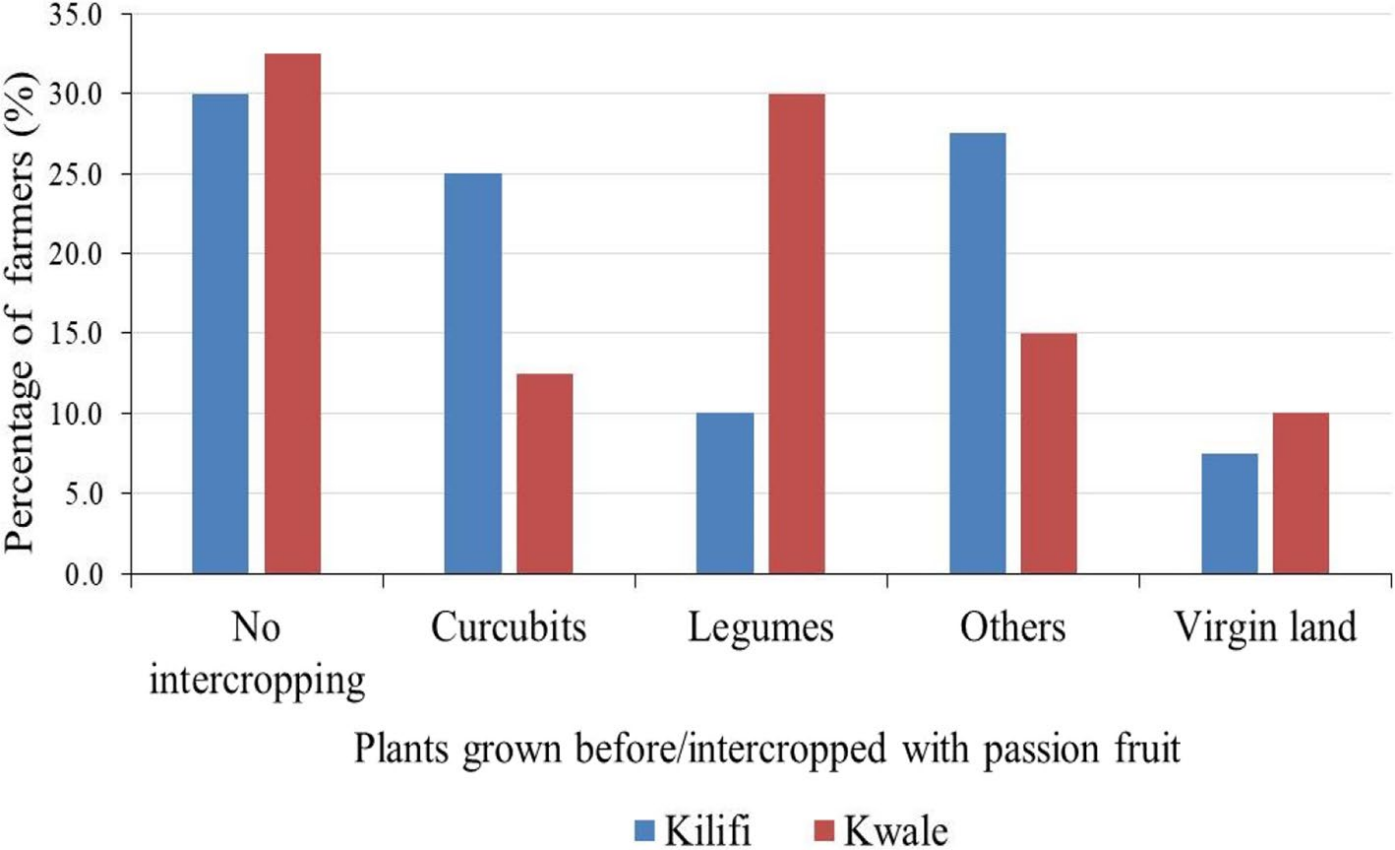
Percentage of farmers growing different crops before planting passion fruit or intercropping with passion fruits in Kwale and Kilifi counties

The highest percentage of farmers in Kilifi (30%) and Kwale (32.5%) counties did not practice intercropping (Fig. 3). However, crops grown before or intercropped with passion fruit differed significantly between the two counties (χ^2^ = 20.696, *p* ≤ 0.05).

### Correlation analysis of management practices and PWD incidence and severity in the different locations of Kwale and Kilifi counties

There was a positive, significant correlation between pruning and disease incidence in Kwale (0.394) and Kilifi (0.423) counties. However, a negative, significant correlation was noted between foliar feeds/ fertilizers and disease incidence (-0.469) in Kwale county. Additionally, a positive, significant correlation between pruning and disease severity was recorded in Kwale county (0.136).

In Kilifi county, there was a positive significant correlation between intercropping and disease incidence (0.312). Table 4 shows a positive significant correlation between disease severity and pruning (0.126), crop rotation (0.161) and intercropping (0.534).

**Table 4:** Correlation analysis of woodiness disease management practices and locations in Kwale and Kilifi counties

County		Management practice						
	Variable	Pruning	Weeding	Pesticides	Irrigation	Foliar feeds	Crop rotation	Inter cropping
Kwale	Incidence	0.394^a^	-0.286	-0.227	-0.065	-0.469^a^	-0.042	0.225
	Severity	0.136^a^	0.079	0.0768	0.054	0.0721	0.0911	0.089
Kilifi	Incidence	0.423^a^	0.159	0.227	0.116	-0.25	0.282	0.312^a^
	Severity	0.126^a^	0.099	0.057	0.024	-0.057	0.161^a^	0.534^b^

^a^Correlation is significant at 0.05 level (2-tailed)

^b^Correlation is significant at 0.01 level (2-tailed)

### Preferred genotypes

The preferred genotypes of passion fruit grown in Kwale and Kilifi counties varied significantly (χ^2^ = 7.671, P < 0.05). In Kwale county, all the farmers preferred yellow passion fruit, while in Kilifi County, 82.5% of the farmers showed a preference for yellow genotype of passion fruit and 7.5% of the farmers showed a preference for KPF 4 genotype of passion fruit. A correlation analysis of the preferred genotype and different locations in Kilifi county revealed no statistical association (χ^2^ = 22.46, *P* > 0.05) (Table 5).

**Table 5:** Proportions of farmers showing preference for different genotypes of passion fruit in Kilifi county

Location	Varieties			
	Yellow	KPF 4	Yellow & KPF 4	Total (%)
Dabaso	9.09	0.00	0.00	7.50
Ganda	6.06	33.33	75.00	15.00
Goshi	6.06	33.33	0.00	7.50
Kijiwetanga	21.21	0.00	0.00	17.50
Mbaraka Chembe	12.12	0.00	0.00	10.00
Mida	12.12	0.00	0.00	10.00
Mkenge	24.24	0.00	0.00	20.00
Mtwapa	9.09	33.33	25.00	12.50

χ^2^ = 22.46, *P* = 0.070

### Correlations between surveyed locations and preferred genotypes

All farmers in Kwale county preferred yellow passion fruit. In Kilifi county, there was a negative correlation between disease incidence and number of farmers growing yellow passion fruit (-1.53). On the other hand, there was a positive correlation between disease incidence and the farmers who showed preference for KPF 4 (0.302) in Kilifi. A positive significant correlation (0.188) was also noted between disease severity and farmers showing preference for both yellow passion and KPF 4 genotypes in Kilifi (Table 6).

**Table 6:** Correlations between surveyed locations and preferred genotypes

County	Responsive variable	Preferred genotypes		
	Disease	Yellow	KPF 4	Yellow and KPF4
Kwale	Disease incidence	-0.130	A	A
	Disease severity	0.072	A	A
Kilifi	Disease Incidence	-0.153	0.302	0.476
	Disease severity	-0.061	0.159	0.188^a^

A = Correlation coefficient could not be computed because at least one of the variables is constant

^a^Correlation is significant at 0.01 level (2-tailed)

**Fig. 4:**
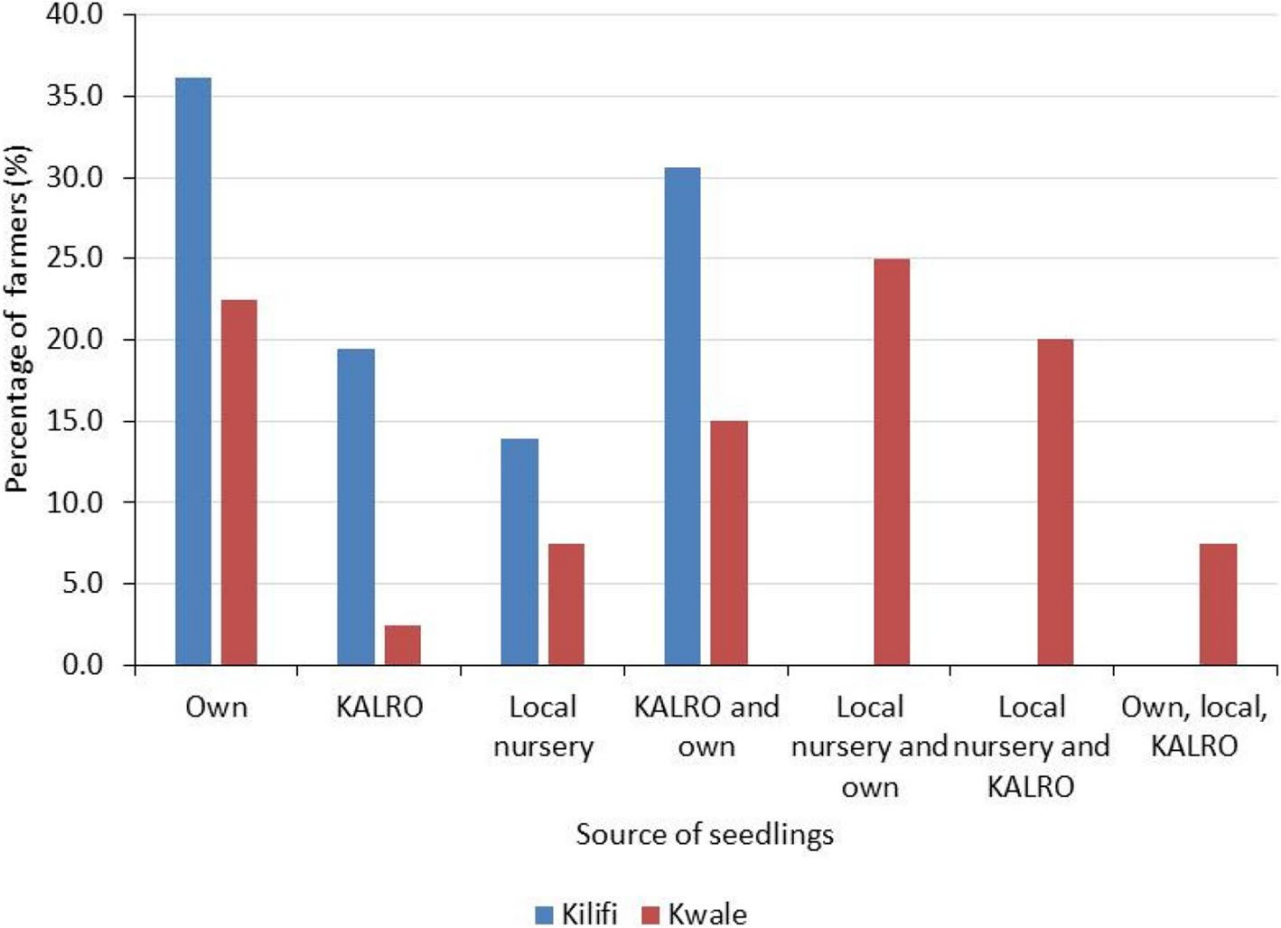
Sources of passion fruit seedlings grown by farmers in Kwale and Kilifi counties

Farmers in Kwale and Kilifi counties obtained their seedlings from different sources which include KALRO, local nurseries, their own nurseries or a combination of sources (Fig. 4). There was a significant difference (χ2 = 51.536, *P* ≤ 0.05) in the source of seedlings in the two counties.

### Greenhouse and field screening of passion fruit against woodiness disease comple

#### Symptomatology in inoculated plants in the greenhouse and field conditions

**Fig. 5:**
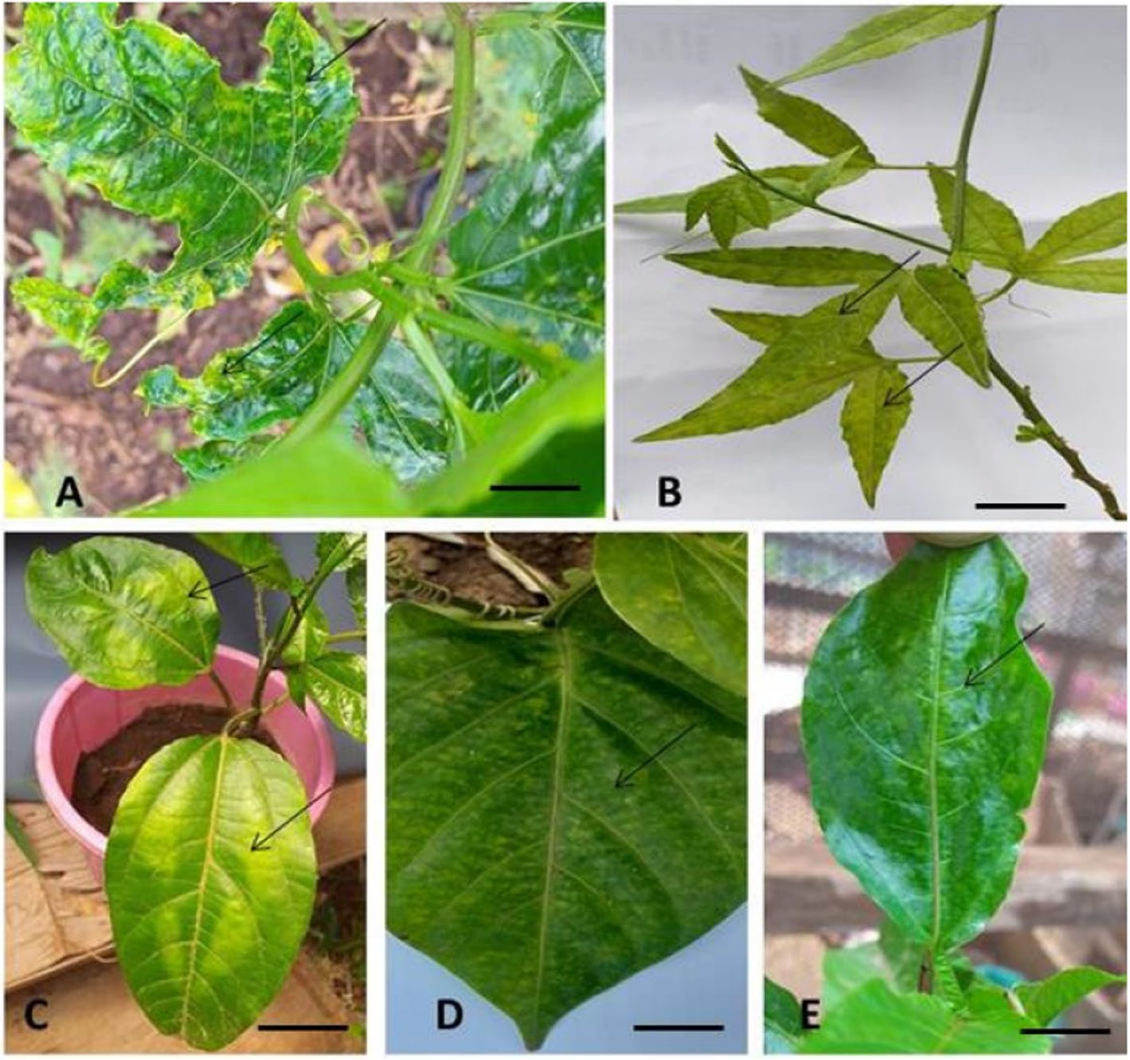
Symptoms of woodiness disease in passion fruit genotypes screened for resistance (Bars = 2 cm). **A**, yellow mosaic, leaf deformation and stunting in purple passion; **B**, leaf mottling in banana passion; **C**, yellow mosaic in KPF 4; **D**, leaf mottling in sweet granadilla; **E**, leaf mottling in yellow passion fruit

Leaves of uninoculated plants both under greenhouse and fields conditions, did not display any symptoms of woodiness disease throughout the evaluation period. Symptoms associated with woodiness disease viruses were observed in inoculated test plants at three weeks post-inoculation. The PWD symptoms observed in all genotypes in both the greenhouse and field were yellow mosaic, leaf mottling, leaf deformation, blister like symptoms and stunting (Fig. 5).

### Disease progression under greenhouse conditions

**Fig. 6:**
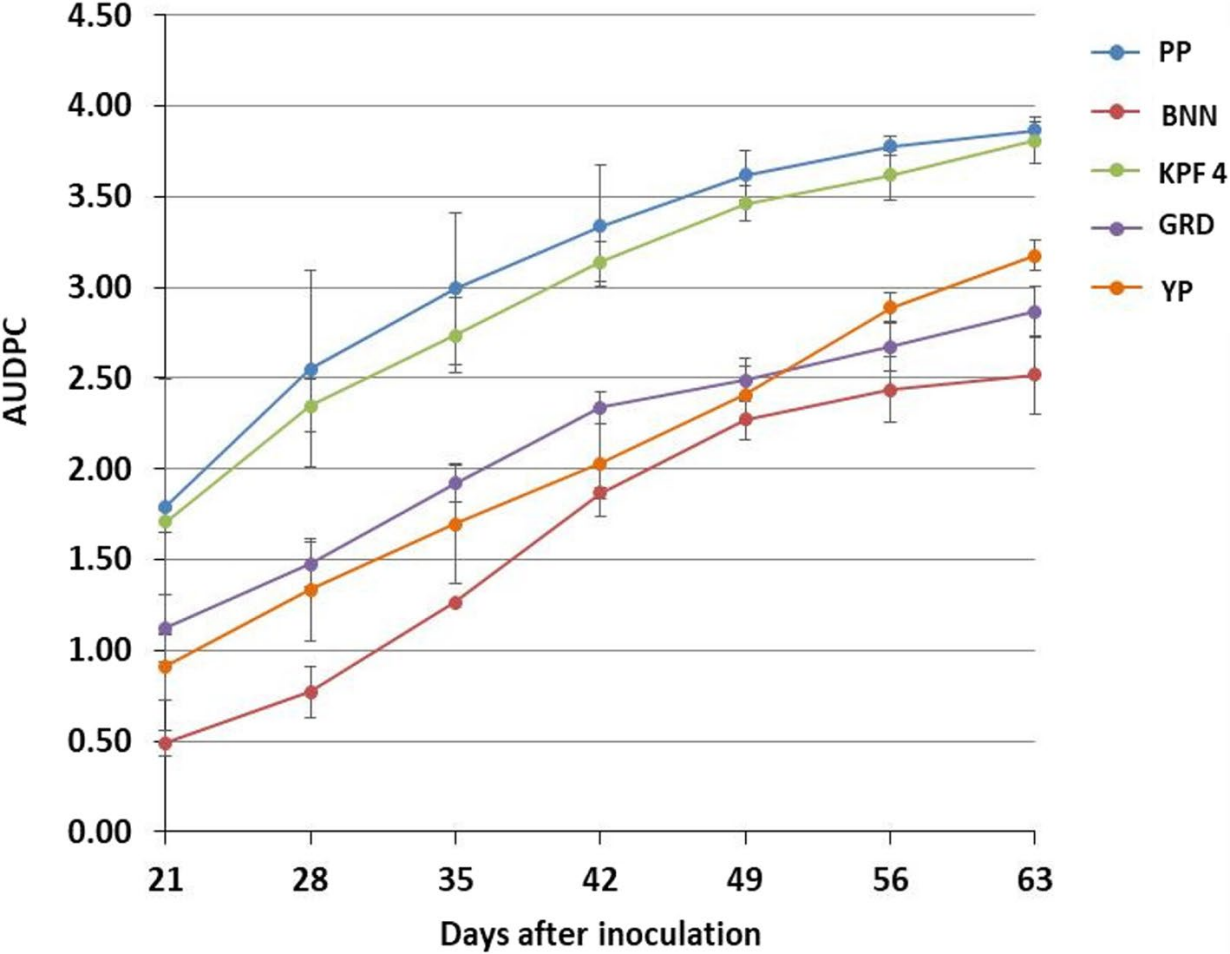
Area under disease progress curve (AUDPC) in passion fruit plants infected with passion fruit woodiness disease virus complexes at different days after inoculation in the greenhouse. PP, purple passion fruit; BNN, banana passion fruit; KPF4, KPF 4 passion fruit; GRD, sweet granadilla passion fruit; YP, yellow passion fruit

The inoculated plants of all tested varieties did not present any characteristic symptom of passion fruit woodiness disease at two weeks post-inoculation. The first PWD symptoms on inoculated plants were observed at third week after inoculation (Fig. 6). The highest disease progression (1.81 to 3.87) was observed on purple passion fruit and the lowest on banana passion fruit (0.5 to 2.52) throughout the evaluation period. Disease progression did not differ significantly (*p* > 0.05) between purple passion fruit and KPF 4 passion fruit. Yellow passion fruit displayed higher AUDPC values (0.9 to 3.18) compared to banana passion (0.5 to 2.52) which were significantly different (*p* ≤ 0.05) on the 28^th^ day and between 56 and 63 days post-inoculation. Disease progression differed significantly (*p* ≤ 0.05) between genotypes KPF 4, purple passion fruit and genotypes yellow passion fruit, banana passion fruit and sweet granadilla.

Based on symptom development, banana passion, sweet granadilla and yellow passion fruits were classified into moderately tolerant while KPF 4 and purple passion fruits genotypes were susceptible (Table 7).

**Table 7:** Grouping of passion fruit genotypes based on symptom development under greenhouse conditions

Genotype	Disease severity range	Classification
Banana passion fruit	1.20—2.33	Moderately tolerant
Sweet granadilla passion fruit	1.40—2.73	Moderately tolerant
Yellow passion fruit	1.33—2.87	Moderately tolerant
KPF 4 passion fruit	1.67- 3.6	Susceptible
Purple passion fruit	1.66–3.66	Susceptible

### Effects of PWD on plant growth under greenhouse conditions

**Fig. 7:**
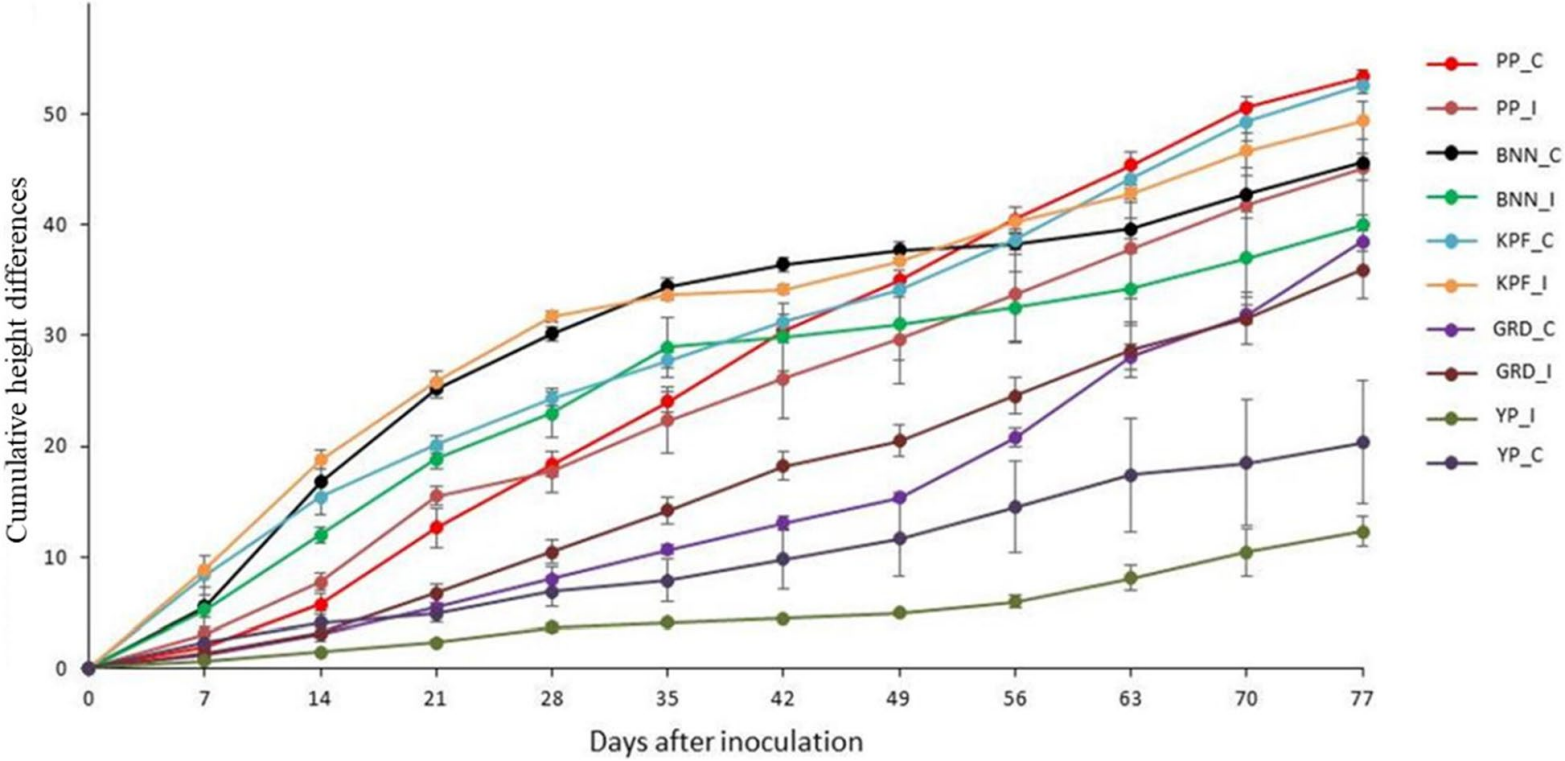
Growth of passion fruit genotypes not inoculated (control) and those mechanically inoculated with passion fruit virus complexes under greenhouse conditions. PP_C (control); PP_I (Infected), purple passion fruit; BNN_C (control); BNN_I (infected), banana passion fruit; KPF_C (control); KPF_I (infected), KPF 4 passion fruit; GRD_C (control); GRD_I (infected), sweet granadilla passion fruit; YP_C (control); YP_I (infected), yellow passion fruit

There was a significant reduction (*p* ≤ 0.05) in plant height of purple passion fruit at 28 days after inoculation after which no significant change in height was recorded throughout the evaluation period (Fig. 7). On the other hand, there was a significant reduction (*p* ≤ 0.05) in height of infected banana passion between 42 and 63 days after inoculation after which a significant increase (*p* ≤ 0.05) was recorded between 70 and 77 days. However, non-inoculated and infected banana passion did not differ significantly (*p* > 0.05) between 42^nd^ and 77^th^ day post inoculation.

The reduction in plant growth of KPF 4 between 35 and 42 days post inoculation differed significantly (*p* ≤ 0.05) between the inoculated and the non-inoculated plants. Although, there was no significant increase (*p* > 0.05) in height of infected sweet granadilla between 21 and 49 days post inoculation that was recorded, there was a significant increase in height (*p* ≤ 0.05) that was recorded between 56 and 77 days after inoculation. No significant difference was recorded in yellow passion fruit throughout the evaluation period.

### Confirmation of passion fruit woodiness disease infection post mechanical sap transmission

All leaf samples from PWD-inoculated passion fruit plants tested positive for potyviruses (Table 8). Potyviruses were not detected in leaf samples from non-inoculated plants. The highest absorbance was recorded in purple passion fruit (0.392) and the lowest absorbance in yellow passion fruit (0.244). Absorbance in yellow passion fruit was significantly (*p* ≤ 0.05) different from all other genotypes except sweet granadilla genotype. The absorbance recorded in non-inoculated plants did not differ significantly (*p* > 0.05) among the different genotypes (Table 8).

**Table 8:** Detection of potyviruses in leaf samples collected from PWD-inoculated plants under greenhouse conditions

Genotype		Absorbance (Abs_405nm_)	ELISA reaction^e^
PP	Non-inoculated	0.090 ± 0.00^d^	-
PP	Inoculated	0.392 ± 0.03^a^	+
KPF4	Non- inoculated	0.093 ± 0.00^d^	-
KPF4	Inoculated	0.332 ± 0.02^ab^	+
BNN	Non- inoculated	0.089 ± 0.00^d^	-
BNN	Inoculated	0.377 ± 0.00^ab^	+
YP	Non- inoculated	0.095 ± 0.00^d^	-
YP	Inoculated	0.244 ± 0.02^c^	+
GRD	Non- inoculated	0.086 ± 0.00^d^	-
GRD	Inoculated	0.302 ± 0.03^bc^	+
Negative control		0.083	-
Positive control (kit)		0.176	+
Positive control (source of inoculum)		0.173	+

*PP* Purple passion fruit, *BNN* Banana passion fruit, *KPF4* Kenya Passion Fruit 4, *GRD* Sweet granadilla passion fruit, *YP* Yellow passion fruit

^e^( +) = positive reaction to the presence of potyviruses; ( −) = negative reaction to the presence of potyviruses. Means having identical letters within the column were not significantly different according to Tukey's HSD test at 5 % level

### Disease progression under field conditions 

**Fig. 8:**
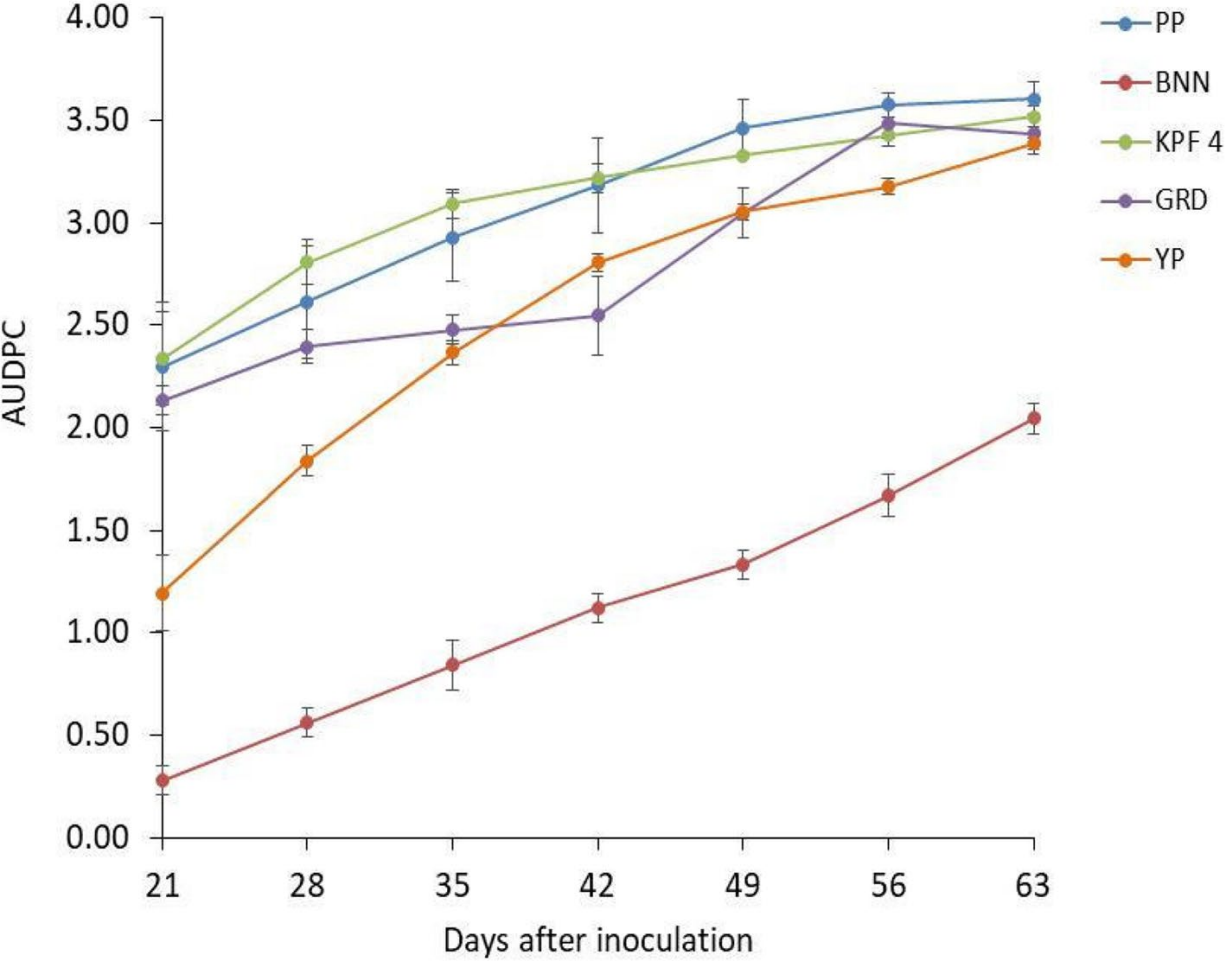
Area Under Disease progress curve (AUDPC) in passion fruit plants infected with passion fruit woodiness disease virus complexes at different days after inoculation under field conditions. PP, purple passion fruit; BNN, banana passion fruit; KPF 4, KPF 4 passion fruit; GRD, sweet granadilla passion fruit; YP, Yellow passion fruit

The first PWD symptoms on inoculated plants were observed at three weeks after inoculation (Fig. 8). The highest AUDPC values (2.34 to 3.22) were observed with KPF4 passion fruit between 21 and 42 days post -inoculation. Purple passion fruit displayed the highest AUDPC values (3.46 to 3.60) between 49 and 63 days post inoculation. However, disease progression did not differ significantly (*p* > 0.05) between purple passion fruit and KPF 4 passion fruit. The disease progression between yellow passion fruit and genotypes KPF4 and purple passion fruit varied significantly between 21 to 35 days post-inoculation. There was a significant difference (*p* ≤ 0.05) between banana passion and rest of the genotypes namely KPF 4, purple passion, yellow passion and sweet granadilla throughout the evaluation period.

Based on symptom development, banana passion fruit genotype was classified into moderately tolerant while sweet granadilla, yellow passion, KPF 4 and purple passion fruit genotypes were susceptible under field conditions (Table 9).

**Table 9:** Grouping of passion fruit genotypes under field conditions based on symptom development

Genotype	Severity range	Classification
Banana passion fruit	1.07—2.13	Moderately tolerant
Yellow passion fruit	1.93—3.13	Susceptible
Sweet granadilla passion fruit	1.4–3.2	Susceptible
KPF 4 passion fruit	2.07—3.27	Susceptible
Purple passion fruit	2.12—3.33	Susceptible

### Effect of PWD on plant growth under field conditions

**Fig. 9:**
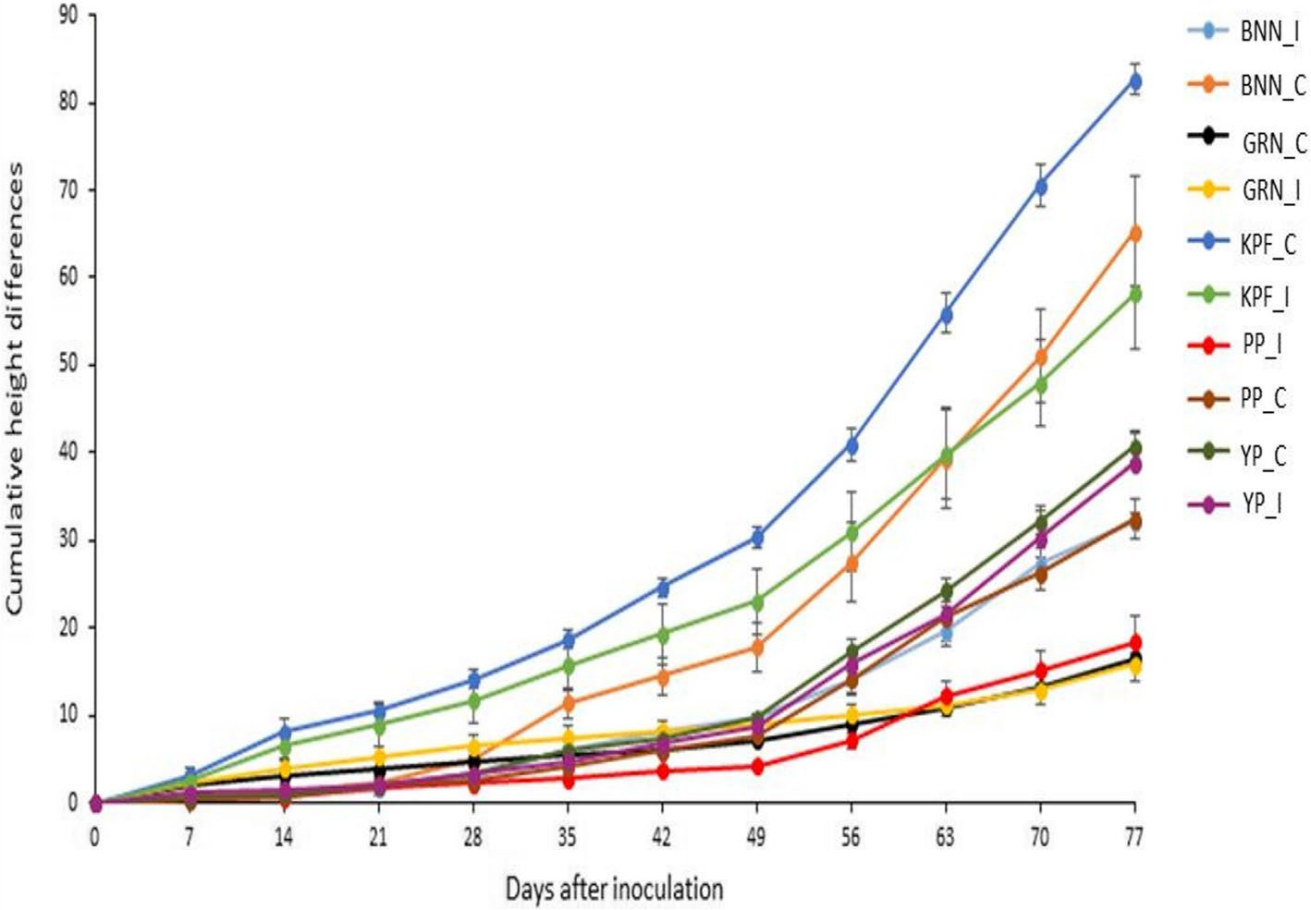
Growth of passion fruit genotypes mechanically inoculated with passion fruit virus complex under field conditions. BNN_C (control); BNN_I (infected), banana passion fruit; GRD_C (control); GRD_I (infected), sweet granadilla passion fruit; KPF_C (control) KPF_I (infected). KPF 4 passion fruit; PP_C (control); PP_I (Infected), purple passion fruit; YP_C (control); YP_I (infected), yellow passion fruit

The increase in plant height in infected banana passion was significantly lower (*p* ≤ 0.05) compared to the non-inoculated plants between 35 to 77 days (Fig. 9). A significant reduction (*p* ≤ 0.05) in plant height was observed between 28 days after inoculation to 49 days post-inoculation. Between 56 and 77 days post-inoculation, a significant (*p* ≤ 0.05) increase in plant growth was recorded. A significant increase (*p* ≤ 0.05) in height was recorded in KPF 4 infected plants. The increase in height in the infected plants of KPF 4, purple passion and banana passion genotypes between 49 and 77 days was significantly (*p* ≤ 0.05) lower compared to the non-inoculated plants.

Although the increase in plant height in purple passion between 35 and 49 days post inoculation did not differ significantly (*p* > 0.05), a significantly lower (*p* ≤ 0.05) growth was recorded in infected purple passion compared to the non inoculated plants between 56 to 77 days post inoculation. On the other hand, increase in height of infected yellow passion fruit plants did not differ significantly (*p* > 0.05) from the non-inoculated plants throughout the growth period.

There was a significant negative correlation between disease severity and plant height in banana passion (-0.787). There was also a negative correlation between disease severity and plant height in KPF 4 (-0.366), purple passion (-0.189), yellow passion (-0.012) and sweet granadilla passion fruit (-0.204) (Table 10).

**Table 10:** Correlation analysis of plant growth and disease severity under field conditions

Experimental site	Responsive variable	Genotypes				
		BNN	KPF 4	PP	YP	SG
Kenyatta University (Main campus)	Plant height	-0.787^a^	-0.366	-0.189	-0.012	-0.204

*BNN* Banana passion, *KPF 4* KPF 4 passion, *PP* Purple passion, *YP* Yellow passion, *SG* Sweet granadilla passion

^a^Correlation is significant at 0.01 level (2 tailed)

### Enzyme linked immunosorbent assay

All leaf samples from PWD-inoculated passion fruit plants tested positive for potyviruses (Table 11). Potyviruses were not detected in leaf samples from non-inoculated plants. The highest absorbance was recorded in purple passion fruit (0.371) while the lowest absorbance was recorded in sweet granadilla passion fruit (0.229). There was no significant difference (*p* > 0.05) in absorbance of inoculated plants in all genotypes tested. There was no significant difference (*p* > 0.05) in absorbance recorded in non-inoculated plants of the different genotypes (Table 11). There was a significant difference between the inoculated passion fruits (PP, KPF4, BNN) and the non-inoculated plants.

**Table 11:** Detection of potyviruses in passion fruit leaf samples collected from plants maintained under field conditions

Genotype		Absorbance (Abs_405nm_)	Potyvirus status^c^
PP	Non- inoculated	0.087 ± 0.00^b^	-
PP	Inoculated	0.371 ± 0.03^a^	+
KPF4	Non- inoculated	0.092 ± 0.00^b^	-
KPF4	Inoculated	0.304 ± 0.03^a^	+
YP	Non- inoculated	0.091 ± 0.00^b^	-
YP	Inoculated	0.259 ± 0.10^ab^	+
BNN	Non- inoculated	0.090 ± 0.00^b^	-
BNN	Inoculated	0.277 ± 0.02^a^	+
GRD	Non- inoculated	0.089 ± 0.00^b^	-
GRD	Inoculated	0.229 ± 0.00^ab^	+
Negative control		0.083	-
Positive control (kit)		0.176	+
Positive control (source of inoculum)		0.173	+

^c^( +) = positive reaction to the presence of potyviruses; ( −) = negative reaction to the presence of potyviruses

*PP* Purple passion fruit, *BNN* Banana passion fruit, *KPF4* Kenya Passion Fruit 4, *GRD* Sweet granadilla passion fruit, *YP* Yellow passion fruit. Means having identical letters within the column were not significantly different according to Tukey's HSD test at 5 % level

## Discussion

### Determination of prevalence, incidence and severity of passion fruit woodiness disease in Kilifi and Kwale counties

Findings from the present study showed that passion fruit woodiness disease (PWD) is widespread with a prevalence of 100% in both Kwale and Kilifi counties in the coastal lowlands of Kenya. This finding is consistent with studies done in Murang’a, Uasin Gishu, Nakuru, Embu, Trans Nzoia, Nyeri, Bungoma, Kirinyaga, Kiambu and Meru counties in Kenya that indicated wide distribution of PWD among farmers’ fields [13]. It has also been reported that PWD is the most widely distributed virus disease infecting passion fruit worldwide [16, 17]. Symptoms observed during surveys in all the locations in Kwale and Kilifi counties included yellow foliar mosaic, leaf mottling, misshapen fruits with corky rinds, leaf curling and distortion, and stunted growth which have been previously reported in Kenya, Uganda, Nigeria and Brazil [13, 16–18].

Viral disease incidence ranged from low (32.50% and 33.75% for Kwale and Kilifi, respectively) to moderate (51.43% and 59.16% for Kwale and Kilifi, respectively). In contrast, very high disease incidence ranging between 70 and 100% has been previously reported in other counties in Kenya including Uasin Gishu, Trans Nzoia, Embu, Kirinyaga, Thika, Meru, Gatundu and Nakuru counties [13]. The difference in PWD incidence in the present study and reports from other regions could be attributed to the different passion fruit genotypes cultivated in the study areas. Kwale and Kilifi counties were dominated by the cultivation of the yellow passion fruit, which is probably moderately tolerant to PWD compared with other passion fruit genotypes including purple and KPF 4 found in other counties.

The disparity in disease incidence could also be due to the difference in agro-ecological zones. Kwale and Kilifi counties are located in the coastal lowlands of Kenya while the counties previously reported with high disease incidences were located in the upper midland and lower highland agro-ecological zones [13] hence different environmental factors which may have had an influence on vector population dynamics and virus transmission rate [19]. Similarly, high incidences (71.8% and 73.1%) in passion fruit vines have also been reported in Brazil [20]. High variations in disease incidence have also been reported in Uganda in the range of 0% to 100% with a mean of 32% [17].

Disease severity in Kwale county ranged from 2.40 to 2.77 while that of Kwale County ranged from 2.65 to 3.18. The difference could be due to the differential susceptibility of the passion fruit genotypes grown in the two counties. In Kwale county, all farms surveyed had the local yellow passion fruit genotype while the farms surveyed in Kilifi county had KPF 4 passion fruit genotype in addition to the largely cultivated yellow passion fruit genotype. In contrast, findings by Kilalo et al. [13] in Uasin Gishu, Nakuru, Embu, Trans Nzoia, Nyeri, Bungoma, Kirinyaga, Kiambu and Meru reported a disease severity in the range of 2.4 to 3.7. This disparity could be due to the different genotypes cultivated in Kwale and Kilifi counties and the counties in the upper midland agroecological zones and the lower highland agro ecological zones [21].

The observed prevalence, incidence and severity levels of PWD in Kwale and Kilifi counties could be exacerbated by poor management practices such as non-sterilization of pruning tools, intercropping with target crops such as cucurbits and cowpeas and crop rotation with the same target crops. According to Fischer and Rezende [15], leguminous crops and cucurbits that harbor passion fruit woodiness virus complex should not be intercropped with passion fruit. In addition, lack of knowledge on proper identification of the woodiness disease symptoms amounts to inability of farmers to rogue infected plants. Use of already infected seedlings obtained from non-certified local nurseries or their own nurseries could also heighten disease incidence and prevalence levels.

### Screening of selected passion fruit genotypes for reactions to PWD under greenhouse and field conditions

Identification of reliable sources of resistance to virus diseases is an important aspect of plant breeding. In this study, under greenhouse conditions, inoculated plants of all the genotypes tested did not display PWD symptoms until three weeks after inoculation. This could be attributed to the low virus replication rates and concentrations in the plants, in addition to the duration required for visual expression of characteristic PWD symptoms. Similar findings were reported by Gonçalves et al. [22], in which no symptoms of *Cowpea Aphid Borne Mosaic Virus* infection were recorded until 20 days after inoculating different genotypes of passion fruit grown in Brazil.

There was significant variation in disease progression in the tested genotypes with purple passion and KPF 4 displaying a higher disease progression compared to yellow passion, sweet granadilla and banana passion fruit genotypes. This shows that purple passion fruit was more susceptible to passion fruit woodiness disease compared to the other passion fruit genotypes. Findings from this study are supported by reports from Cerqueira-Silva et al. [23] indicating varying susceptibilities of different passion fruit varieties to woodiness disease and a broad genetic variability for *Passiflora edulis* and wild *Passiflora* species. This probably shows that the variation in disease progression could be due to the genotypic effect. Although disease progression was observed in all the tested genotypes, plants of banana passion fruit (BNN) showed delayed symptom expression with the first symptoms appearing in most of the plants at 35 days post-inoculation. The delayed symptom observed in banana passion fruit may be due to the presence of resistance genes which are providing a certain degree of PWD resistance.

Plants of all the tested passion fruit genotypes inoculated with PWD exhibited significantly reduced growth compared to the non-inoculated plants. These results clearly indicate the high potential damage that passion fruit woodiness disease (PWD) can cause to passion fruit plants, especially when young plants are infested. These findings are consistent with previous report by Karani-Gichimu et al. [4], indicating that passion fruit woodiness disease affects plant growth and lifespan of passion fruit vines.

Passion fruit genotypes varied greatly in the symptoms caused by PWD and in the severity of symptom expression in the field. The Area Under Disease Progress Curve (AUDPC) for the five passion fruit genotypes varied significantly among them. The differential susceptibility to PWD may be due to the genetic variability within the screened genotypes as reported by Cerqueira-Silva et al. [24] and Freitas et al. [25]. Studies conducted by Schuerger and Hammer [26] also revealed that the genetic background might influence the apparent relative effectiveness of the resistant genes of the plant, resulting in many genotypes becoming susceptible to a virus attack.

ACP ELISA assays using universal potyvirus antiserum (Agdia Inc., Elkhat, IN) confirmed that the observed characteristic symptoms of woodiness disease were as a result of potyvirus infection. This is supported by the findings of Fukumoto et al. [27] and Cerqueira-Silva et al. [28] describing different types of potyviruses as potential pathogens of passion fruit woodiness (PWD) in Africa, Asia and Brazil.

## Conclusions

Passion fruit woodiness disease is widespread in Kwale and Kilifi counties with low to moderate disease incidence and severity. In addition, the study established that the response of passion fruit genotypes to woodiness viruses was genotype dependent. There is need to sensitize farmers on the cause and spread of PWD and management strategies in order to increase production and enhance the quality of fruits.

## Materials and methods

### Description of study sites

**Fig. 10:**
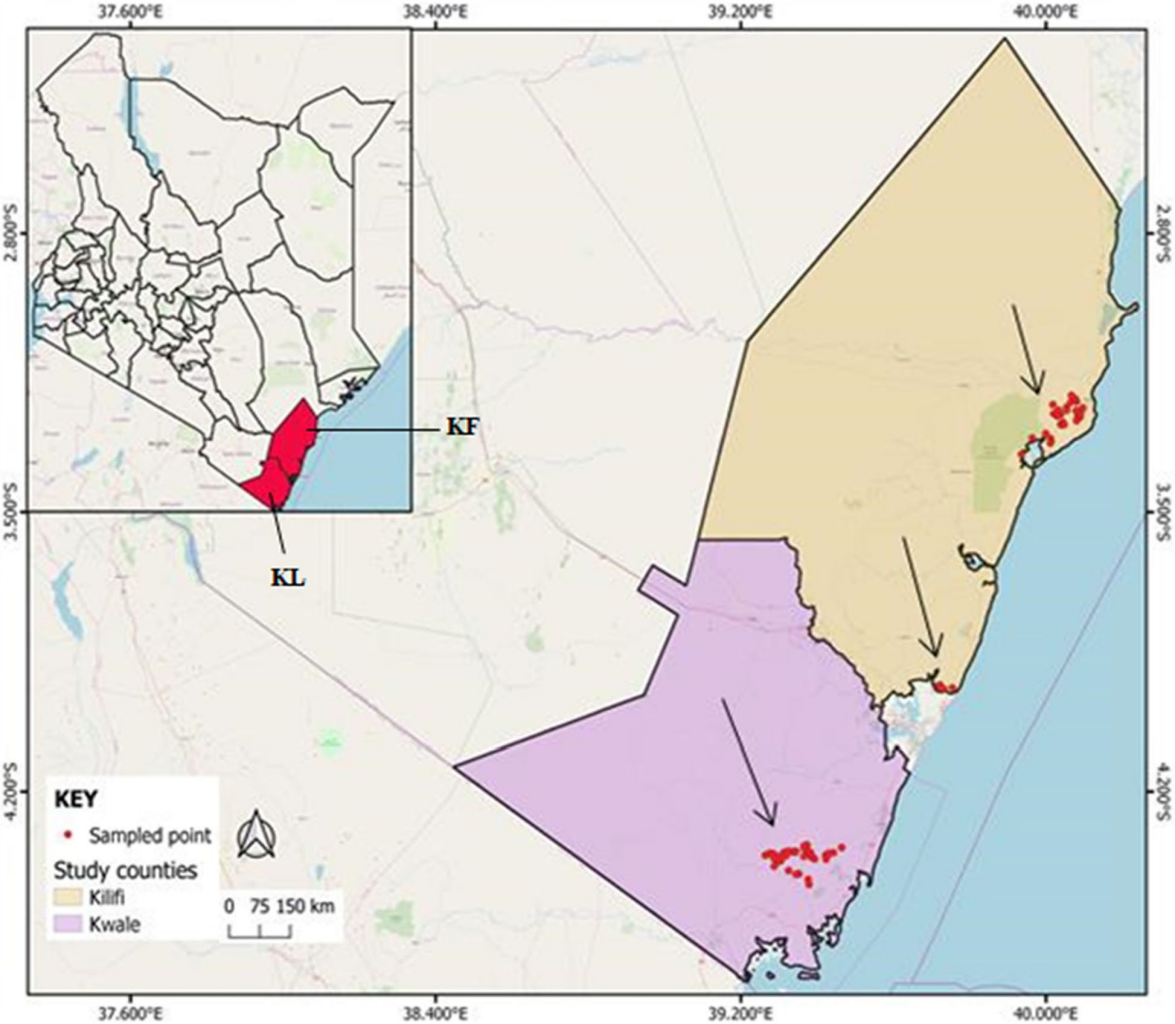
A map of Kenya displaying Kilifi (KF) and Kwale (KL) counties where the survey was undertaken. Red points indicate sampled orchards ( © *QGIS 2020 software v*ersion 3.14.0 -Generated by Asande)

The study sites were located in Kwale (CL3) and Kilifi (CL4) counties in the coastal region of Kenya (Fig. 10). Kwale county borders Indian Ocean to the East, Tanzania to the South West, Taita Taveta to the West, Kilifi to the North and Mombasa to the North East. The county lies within a longitude of 38° 27' E and 39° 40' E, latitude of 3° 30’ S and 4° 40' S and an altitude of 0 to 462 m above sea level. The county receives rainfall in the range of 900 mm and 1500 mm per annum with a bimodal distribution pattern and temperatures in the range of 22 °C to 34 °C [29]. The area is also characterized by loamy, clay and sandy soils [30].

Kilifi county on the other hand, borders Indian Ocean to the East, Kwale county to the South West, Taita Taveta county to the West, Mombasa county to the South and Tana River county to the North. It lies between a latitude of 2° 20' S and 4° 0' S, longitude of 39° 05' E and 40° 14' E and altitude ranges from 0 to 450 m above sea level. The county receives rainfall in the range of 400 mm to 1,300 mm with a bimodal distribution pattern and a mean annual temperature of about 27 °C. Additionally, Kilifi county is characterized by well drained, fine sandy loam to fine sandy clay loam soils [31].

Greenhouse and field experiments to evaluate resistance of passion fruit genotypes to woodiness disease were carried out between September and December, 2019 at the Department of Plant Sciences, Main campus, Kenyatta University. The University is located between Nairobi and Thika at approximately 20 km by road from Nairobi city at an altitude of 1608 m above sea level and longitude of 36° 55′ 0E and latitude of 1° 10′ 60S. The area receives rainfall range of between 1000—1100 mm with a bimodal distribution pattern while temperatures range between 12 °C and 24.6 °C. The site is in upper midland agro-ecological zone 1 (UM3). The area is characterized by dark reddish brown to dark brown loam soils.

### Selection of survey study sites and sampling procedure in farmers’ fields

A survey study was carried out in passion fruit orchards in major growing areas in Kwale and Kilifi counties. The specific locations of the survey were chosen in consultation with research scientists from Kenya Agricultural and Livestock Research Organization (KALRO), located in Matuga, Kwale county and Msabaha, Kilifi county. The survey area encompassed administrative locations where groups of orchards were found.

Selected farms were at least 1 km apart and each farm contained a minimum of 100 passion fruit plants aged between 6 months and 3 years. A sample size of 40 passion fruit orchards per county was determined in accordance with the formula by Mugenda and Mugenda [32]. Purposive sampling was conducted and disease assessment was carried out.

A questionnaire was administered to 80 passion fruit farmers to capture background information, their knowledge and experience with passion fruit woodiness disease (PWD) and document how they address the problem. Information on farm size, passion fruit varieties planted, preferred varieties, source of passion fruit seedlings, farm management practices, types of pests and other diseases affecting passion fruit was captured using the Online Data Kit software on a smart phone. Prior to the formal data collection, the questionnaire was pre-tested on a small group of farmers and adjustments were made to ensure validity and clarity of the content.

### Disease assessment and data collection in the field

Passion fruit woodiness disease assessments were conducted between September and November 2019. Fields were randomly sampled at 1 km intervals on the main and rural accessible roads. On a 50 × 50 m area, an examination of the farm was carried out diagonally at random and diseased plants were counted along the two diagonals according to Kilalo et al. [13]. Disease incidence was obtained by calculating the ratio of the diseased plants with PWD symptoms to the total number of plants assessed expressed as a percentage [33]. Disease severity was also determined in all orchards surveyed. Plants were evaluated for woodiness disease symptoms using a five category scale where, 1 = absence of infection, 2 = mild infection, leaf deformation; 3 = moderate infection, leaf deformation and stunting; 4 = severe infection and stunting; 5 = very severe infection, severe stunting and plant death [13]. Disease prevalence was obtained by calculating the ratio of the fields with disease symptoms to the total number of fields assessed expressed as a percentage [34].

### Evaluation of selected passion fruit genotypes for resistance to passion fruit woodiness disease

#### Sources and types of passion fruit genotypes used for assessment of resistance to woodiness disease

**Fig. 11:**
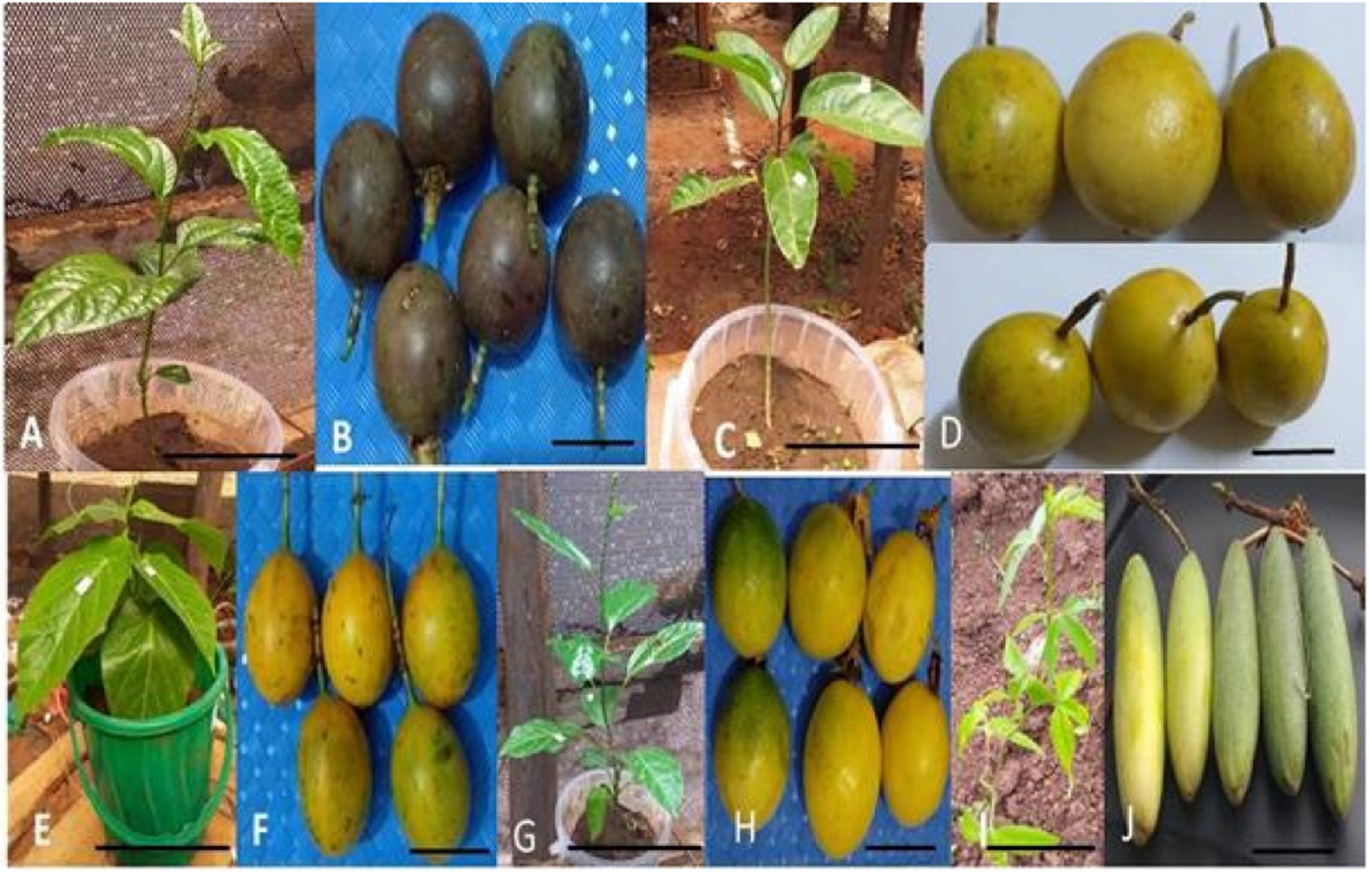
Passion fruit varieties screened for resistance against woodiness disease. **A**, Purple passion (*Passiflora edulis* f*. edulis*) vine (Bar = 10 cm); **B**, Purple passion (*Passiflora edulis* f*. edulis*) fruits (Bar = 2 cm); **C**, Yellow variety (*Passiflora edulis* f*. flavicarpa*) vine (Bar = 10 cm); **D**, Yellow variety (*Passiflora edulis* f*. flavicarpa*) fruits (Bar = 2 cm); **E**, Sweet granadilla (*Passiflora ligularis*) vine (Bar = 10 cm); **F**, Sweet granadilla (*Passiflora ligularis*) fruits (Bar = 2 cm); **G**, KPF 4 vine (Bar = 10 cm), a cross between *Passiflora edulis* f*. edulis* and *Passiflora edulis* f*. flavicarpa* (Ssemwanga, 2007); **H**, KPF 4 fruits (Bar = 2 cm); **I**, Banana passion (*Passiflora mollissima*) vine (Bar = 10 cm); **J**, Banana passion (*Passiflora mollissima*) fruits (Bar = 2 cm)

Five different passion fruit genotypes (Table 12) namely purple passion, yellow passion, sweet granadilla passion, Kenya Passion Fruit 4 (KPF 4) and banana passion (Fig. 11) were acquired from Jomo Kenyatta University of Agriculture and Technology (JKUAT), KALRO (Thika) and KALRO (Mtwapa). The fruit genotypes were selected based on the species of passion fruit cultivated in Kenya and also breeders’ lines. The purple passion fruit was used as a susceptible control. There was no resistant passion fruit genotypes used in this study since there is no information available on the level of resistance of Kenyan passion fruit germplasm. The passion fruit genotypes were screened under field and greenhouse conditions at Kenyatta University, Kenya.

**Table 12:** Sources and types of passion fruit genotypes used for assessment of resistance to woodiness disease

Source	Genotype	Species	Characteristics
KALRO (Thika)	Purple passion Fruit	*Passiflora edulis* f. *edulis*	Round-shaped fruits with a purple rind, pulp is rich in aroma and flavor, less acidic compared to yellow passion fruit, susceptible to PWD
KALRO (Mtwapa)	Yellow passion Fruit	*Passiflora edulis* f*. flavicarpa*	Resistant to *Fusarium* wilt and nematodes, fruits have yellow rind with an acidic flavor, response to PWD inoculation not known
JKUAT	Sweet granadilla	*Passiflora ligularis*	Orange to yellow fruit colour with lesser light markings, round-shaped fruits with a tip that ends in the stem, response to PWD inoculation not known
JKUAT	KPF 4	Hybrid (*Passiflora edulis* f. *edulis* × *Passiflora edulis* f. *flavicarpa*)	Drought tolerant, yellow and sweet fruit with high juice content, farmer-preferred variety, response to PWD inoculation not known
KALRO (Thika)	Banana passion	*Passiflora mollissima*	Yellow and oblong–shaped fruit, sweet fruits with a characteristic flavor, response to PWD inoculation not known

Source: NAFIS (2008)

## Source and preparation of inoculum

Symptomatic fruits and leaf tissues from diseased plants were collected in Kwale and Kilifi counties. The fruit and leaf tissues from Kwale county were used for screening passion fruit genotypes against PWD. The diseased leaves and fruits were ground in the presence of 0.05 M potassium phosphate (1 g in 5 ml) buffer pH 7.0 and the extracts were filtered through cheese cloth. A small quantity (1.0 g) of carborundum (600 mesh) was added to the plant extracts having the virus [16]. The extracts were then used to inoculate healthy plants both under greenhouse and field conditions at Kenyatta University, Kenya.

### Greenhouse based evaluation

Seedlings of different passion fruit genotypes were planted in 5-L plastic pots comprising of top soil well melded with farmyard manure (3:1). At planting, 10 g of Diammonium phosphate (DAP) was also applied per individual seedling. A completely randomized design was used to lay out the experiments. Each treatment had 20 plants (one plant per pot) and was replicated 3 times.

### Plant inoculation with passion fruit woodiness virus complex

Prior to inoculation, the plants were screened for potyviruses through indirect ACP ELISA to ensure they were free from infection. The first two leaves of healthy plants (8 to 12 weeks old) were mechanically inoculated through conventional leaf rub method [22]. The control was not inoculated with the virus. Instead, 20 plants per genotype were inoculated with the extraction buffer alone as negative controls.

**Fig. 12:**
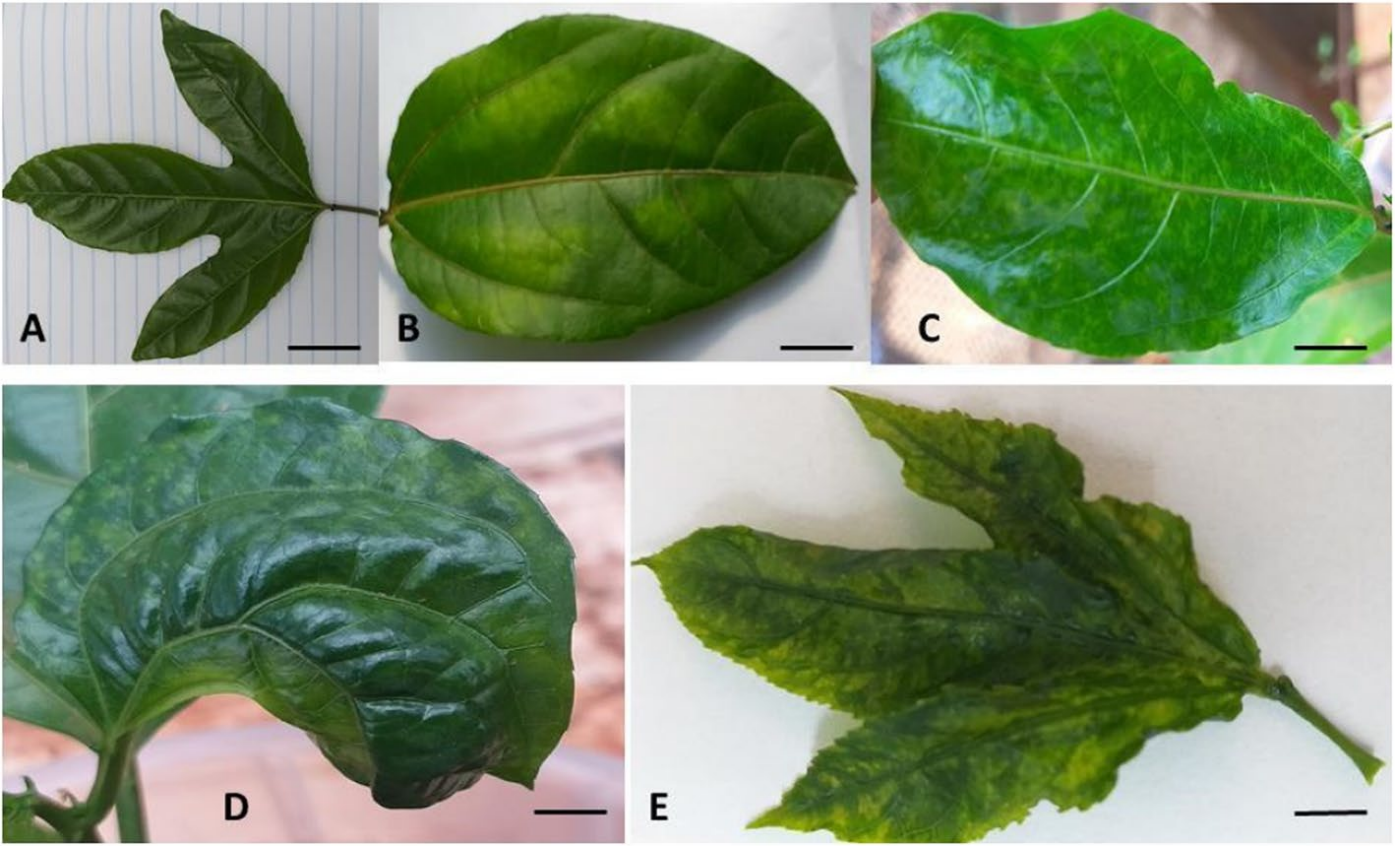
Disease severity category scale (Bars = 1 cm). **A**, 1 = absence of infection; **B**, 2 = mild infection; **C**, 3 = moderate infection; **D**, 4 = Severe infection; **E**, 5 = very severe infection, severe mottling, deformation

Inoculations were repeated twice at a seven-day interval and the plant responses were observed for three months. Watering (500 ml per plant) was carried out once a day, in the morning. The plants were protected from pests using Emamectin benzoate 19 g L^−1^ w/v and Sulpher 500 g L^−1^ w/v to prevent spread of the disease to other plants. Sulpher 500 g L^−1^ w/v was also effective in controlling fungal diseases. Reaction of different genotypes to the potyviruses was monitored and scored on a weekly basis. Symptom severity on passion fruit plants was scored beginning 1 week after inoculation on a category scale of 1—5 by visual examination of the disease symptoms on specific plants where; 1 = absence of infection, 2 = mild infection, leaf deformation, 3 = moderate infection, leaf deformation and stunting; severity 4 = severe infection and stunting and 5 = very severe infection [13] (Fig. 12).

At the end of the experiment, data obtained using the disease severity scale was used to compute the Area Under the Disease Progress Curve (AUDPC) [3] for every single genotype evaluated.

### Field based evaluation

#### Field preparation and experimental design

Field plots established at Kenyatta University Main campus were cleared and ploughed to obtain a fine tilth. A randomized complete block design was used to lay out the experiment. Five different passion fruit genotypes were planted in holes of 45 cm × 45 cm × 45 cm. Each plot had 4 rows with 5 plants per row. A spacing of 1 m between rows and 1.5 m within rows was maintained. The plots were 1 m apart. Each experimental plot had a single genotype and was replicated three times. There was an additional plot per variety without treatment (control).

Each planting hole was filled with topsoil well mixed with 1 kg of farmyard manure. At planting, 10 g of Diammonium phosphate (D.A.P, 18–46-0) was also applied per individual seedling.

### Inoculation of healthy passion fruit plants with woodiness virus complex

Prior to inoculation, the plants were screened for potyviruses through indirect ACP ELISA to ensure they were free from infection. The first two fully expanded leaves of healthy plants (8 to 12 weeks old) were mechanically inoculated using the conventional leaf rub method. The control plants were inoculated with the extraction buffer. Inoculations were repeated as described in the greenhouse experiment above. Plants were also maintained in the field as described in the greenhouse. Assessment of disease resistance was carried out, based on visual symptoms of the disease and by comparing the rate of symptom development which includes leaf mosaic, distortion and reduction in size. Disease severity based on symptoms was assessed per variety using the five category scale as described in the greenhouse experiment. At the end of the experiment, data obtained by the severity scale and plant height was recorded as described in the greenhouse.

Data obtained using the disease severity scale was used to compute the Area Under the Disease Progress Curve (AUDPC) [35] for every single genotype evaluated.

### Enzyme-linked immunosorbent assay

The presence of PWD in passion fruit plants inoculated with PWD was ascertained through indirect ACP- Enzyme Linked Immuno-sorbent Assay (Agdia Inc., Elkhart, USA). Three leaves of each of the inoculated and control plants of passion fruit genotypes were collected 12 weeks after mechanical inoculation with potyviruses. The samples were assayed based on monoclonal antibodies (PTY 1) through ACP—Enzyme Linked Immuno-sorbent Assay as per Agdia's Potyvirus Group test. Crude leaf extracts were prepared from leaves of potyvirus inoculated and mock inoculated plants by grinding in an indirect sample extraction buffer (1 g in 100 ml) using a clean mortar and pestle.

A 100 μl of each sample extract was dispensed into the sample wells in an empty microtitre plate. A 100 μl of the positive control was also dispensed in two empty wells. Similarly, a 100 μl of the sample extraction buffer (IEB) was loaded into two empty wells. The plate was then set in a humid box and incubated for 1 h at room temperature (18–30 ºC). All the enzymes conjugates and antibodies were prepared as per the manufactures instructions. When the incubation period was complete, the microtitre plate was cautiously washed using the wash buffer (1 × PBST) and tapped firmly on paper towels. This procedure was repeated 8 times. Subsequently, 100 μl of prepared detection antibody (dissolved in 1 × ECL buffer) was dispensed to each well. The plate was set in a humid box and incubated overnight at 4 °C. After incubation, the plate was again washed 8 times with 1 × PBST (wash buffer). The plate was tapped firmly to remove excess buffer and bubbles. A 100 μl of alkaline phosphatase enzyme conjugate (dissolved in 1 × ECL buffer) was added per well. The plate was again incubated for 1 h at room temperature. After incubation, the plate was again washed thoroughly 8 times with 1 × PBST. The plate was tapped firmly on paper towels to remove excess buffer and air bubbles.

Lastly, 100 μl PNP substrate was added into each well and the plates were incubated for 60 min. The plates were covered with aluminium foil to protect them from direct and intense light. The wells were visually examined and optical density values read using a plate reader at 405 nm. Samples with optical density values greater than twice the average of negative controls, at 405 nm, were deemed positive. The positive controls (Inoculum) were used to confirm that the experiment worked.

### Data analysis

Pearson correlation, Pearson Chi-square and cross tabulation in SPSS statistics 20 were used to analyze data from the questionnaire. A logarithmic transformation (Log_10_ X, where X = severity score) was applied to the data on severity and then subjected to Analysis of Variance (ANOVA) to assess significant differences and separation of means carried out using Tukey’s honest significant difference test at 5% level. The data was analyzed using Genstat 15^th^ Edition statistical program.

Data obtained from the five category grading scale was used to compute Area Under Disease Progress Curve (AUDPC) [35] in all genotypes assessed, according to the expression;

$$AUDPC=\sum_{\mathrm{i}=1}^{n-1}\left(\frac{{y}_{i }+ {y}_{i+1}}{2}\right)\left({\mathrm{t}}_{\mathrm{i}+1}-{\mathrm{t}}_{\mathrm{i}}\right)$$

y_i_ = disease level at time t_i_.

t_i+1_—t_i_ = Time (days) between two disease scores.

### Supplementary Information

**Additional file 1: Appendix I.** Questionnaire.

## Data Availability

All data generated or analysed during this study are included in this published article.
