# Supplementary material for: Occurrence of passion fruit woodiness disease in the coastal lowlands of Kenya and screening of passion fruit genotypes for resistance to passion fruit woodiness disease
Source: BMC Plant Biol. 2023 Nov 6;23:544. doi: 10.1186/s12870-023-04546-8 (PMC10626802; doi:10.1186/s12870-023-04546-8)
Supplement: Supplementary file 1 — Additional file 1: Appendix I. Questionnaire. [file 12870_2023_4546_MOESM1_ESM.pdf]

APPENDICES

Appendix I: Questionnaire

Interviewer: Lydia Asande, a PhD student at Kenyatta University

I am carrying out a survey on the incidence and severity of passion fruit woodiness disease in coastal Kenya for intervention purposes. Kindly respond to the questions below. The information you will give will remain confidential and will only be used for the research purposes.

1. Details of the farmer

(a) Name of the farmer (Optional)____________________

(b) Gender____________________

(c) Occupation________________________

2. Location details

GPS reading__________ Total farm acreage______________________

County_______________________ Division_________ Location___________ Sub location ___________

Altitude_______________

Topography: 1 = Steep slope 2 = Gentle slope 3 = Valley 4= Hill top 5 = flat

1. List the varieties of passion fruit in your farm

2. How old are your plants?________________

3. What is the total number of plants in your farm?___________

4. For how long have you grown passion fruits?__________

5. Name your preferred varieties________________________________

Reason____

6. What is the source of your passion frut seedlings?
   1 = Your own

   2 = Local nursery

   3 = Research Institution

   4 = Other (specify)

7. Do you practice
   a) Small scale farming
   b) Large scale farming

8. Is it (a) your own enterprise (b) contracted

9. What crop were you growing before shifting to passion fruit?______________________

10. Is there an incidence of passion fruit woodiness disease in your farm? [Yes] [No]

If yes, how would you rate the infection

1= Wide spread

2=Scanty

11. How do you control woodiness disease in your farm?

12. Tick the maintenance practices of passion fruit carried out in your farm
    a) Pruning

    b) Weeding [Manual] [herbicides]

    c) Spraying with pesticides

    d) Mulching

    e) Irrigation

    f) Application of foliar feeds or fertilizers

g) Crop rotation

Other (specify)___________________________

If you practice pruning, do you sterilize your apparatus? [Yes] [No]

If yes, how do you sterilize them?

10. Do you practice intercropping?

If yes, with which crops in order of preference

13. What other diseases do you experience in your farms

14. Are pests a problem in your farm? [Yes] [No]

a) If yes how do you control them?

b) List the pests

15. What are the other challenges of passion fruit farming do you face?
